# Ataxin-3, DNA Damage Repair, and SCA3 Cerebellar Degeneration: On the Path to Parsimony?

**DOI:** 10.1371/journal.pgen.1004937

**Published:** 2015-01-29

**Authors:** Jacqueline M. Ward, Albert R. La Spada

**Affiliations:** 1 Biomedical Sciences Graduate Program, University of California, San Diego, La Jolla, California, United States of America; 2 Departments of Pediatrics, Cellular & Molecular Medicine, and Neurosciences, Division of Biological Sciences, the Institute for Genomic Medicine, and the Sanford Consortium for Regenerative Medicine, University of California, San Diego, La Jolla, California, United States of America; 3 Rady Children’s Hospital, San Diego, California, United States of America; The Hospital for Sick Children and University of Toronto, CANADA

Spinocerebellar ataxia type 3 (SCA3), also known as Machado-Joseph disease, is an autosomal dominant neurodegenerative disorder, characterized by progressive ataxia, spasticity, and ocular movement abnormalities. The SCA3 gene, *ATXN3*, was identified in 1994 and contains a CAG repeat tract that increases from 12–41 to 62–84 in the disease state to produce ataxin-3 protein containing a polyglutamine (polyQ) expansion [1]. SCA3 is the most common inherited ataxia, yet a thorough understanding of the molecular basis of this disease has remained elusive. Initial studies found that ataxin-3 interacts with two proteins, HHR23A and HHR23B, that are both homologs of the DNA repair protein Rad23 [2]. Further work revealed ataxin-3 as a bona fide deubiquitinating enzyme [3,4], thereby providing a crucial insight into ataxin-3’s normal function. More recently, ataxin-3 was shown to interact with the transcription factor FOXO4 as a transcriptional coactivator in the oxidative stress response [5]. Thus, while some very basic functions of ataxin-3 have been established, these advances have yielded nonintersecting lines of investigation and have not provided an underlying mechanism for SCA3 disease pathogenesis.

**Figure 1:**
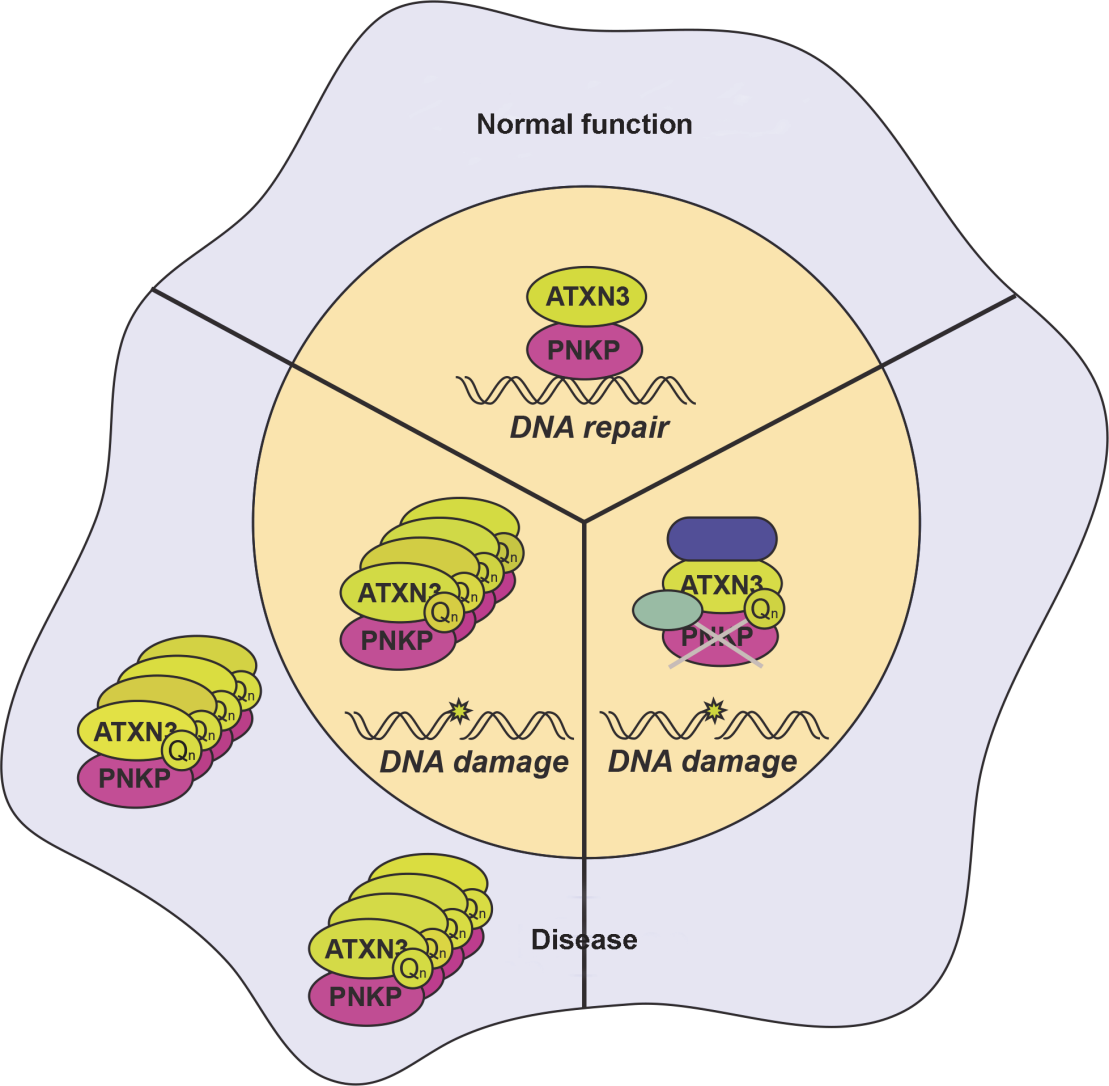
Interaction of ataxin-3 and PNKP in normal and disease states. ‘Normal’ function of the ataxin-3–PNKP interaction is to coordinately promote DNA repair (top). However, in SCA3 ‘Disease’, polyglutamine-expanded ataxin-3 may sequester PNKP in aggregates to prevent PKNP-mediated DNA repair activity (lower left). Alternatively, in SCA3 ‘Disease’, polyglutamine-expanded ataxin-3 may prevent PKNP DNA repair function through an aggregate-independent mechanism by interacting with PKNP in its native complex to inhibit it (lower right).

In this issue of *PLOS Genetics*, two complementary papers establish a key function of ataxin-3 through its interaction with the DNA end-processing enzyme polynucleotide kinase 3′-phosphatase (PNKP) and reveal how this interplay contributes to SCA3 pathogenesis [6,7]. The work by Chatterjee et al. uncovers the interaction between ataxin-3 and PKNP in a screen for PNKP-associated proteins. PNKP is a 3′ DNA phosphatase involved in repair of single- and double-strand breaks through the single-strand break repair and nonhomologous end joining pathways. Interestingly, mutations in *PNKP* have been linked to disorders with neurological symptoms including neurodegeneration, progressive polyneuropathy, cerebellar atrophy, microcephaly, mild epilepsy, seizures, developmental delay, and intellectual disability [8,9]. PNKP is an enzyme with key roles in DNA damage repair, particularly maintaining genomic stability of neural cells, and in providing resistance of cancer cells to genotoxic therapeutic agents. To this end, PNKP-targeting drugs are being developed with the goal of potentially increasing the sensitivity of human tumors to gamma radiation [10]. Chatterjee et al. show that ataxin-3 is a robust interactor with PNKP and promotes PNKP’s phosphatase and DNA repair activities (Fig. 1). Importantly, expression of mutant ataxin-3 leads to decreased phosphatase activity in vitro and in the brains of SCA3 transgenic mice.

The accompanying work from Gao et al. shows that PNKP also interacts with polyQ-expanded ataxin-3. Interestingly, the PNKP–ATXN3 interaction predominantly occurs outside of the nucleus in SCA3 patient brains, and the authors document colocalization of PNKP with polyQ-containing aggregates in SCA3 patient brains. Contrary to the nuclear inclusions observed by other groups, these aggregates appear outside of the nucleus and suggest that polyQ-ataxin-3 aggregates may sequester PNKP in the cytoplasm, resulting in a loss of normal nuclear PNKP function. Further work will need to delineate this possibility and further explore this unexpected phenomenon.

Gao et al. also demonstrate a dramatic increase in DNA damage in mutant ataxin-3 cells, SCA3 mouse brain, and brain sections from SCA3 patients, corroborating results observed in the Chatterjee et al. paper. Importantly, the authors link this increase in DNA damage to activation of the ataxia-telangiectasia-mutated (ATM) signaling pathway. Previous work had shown mutant ataxin-3 results in p53 activation and apoptotic cell death [11]. The current work corroborates this earlier finding and documents that this effect can be rescued by PNKP overexpression, implicating loss of PNKP activity in the path of p53-mediated apoptosis. In a separate tack, Gao et al. implicate the c-Abl/PKCδ arm of the ATM signaling pathway by illustrating PKCδ phosphorylation and nuclear translocation.

While these two papers clearly connect PNKP to SCA3 pathogenesis, remaining work will need to clarify this relationship. In many experiments, expression of polyQ-ataxin-3 had the same effect as depletion of either ATXN3 or PNKP. It seems clear that mutant ataxin-3 is responsible for the loss of PKNP function, but can the proposed sequestration of PNKP in polyQ-ataxin-3 aggregates fully account for failure of DNA damage repair (Fig. 1)? Studies of other polyQ diseases suggest that interactions in the soluble phase are an important aspect of the pathogenic cascade; hence, it is also possible that mutant ataxin-3 deleteriously interacts with PNKP independent of aggregate sequestration (Fig. 1). Although Chatterjee et al. found that polyQ-ataxin-3 does not interact with the phosphatase domain of PNKP in vitro, could mutant ataxin-3 nonetheless interact with PNKP in its native complex to inactivate its phosphatase activity? Interaction of polyQ disease–associated proteins in their normal native complexes has emerged as a likely scenario for altering the functions of presumed interactors in SCA1 and spinobulbar muscular atrophy [12,13], two related polyQ disorders.

The identification of an interaction between PNKP and ataxin-3 is intriguing, as there were previous hints from the SCA3 field that DNA damage might be involved in this disease. One of the first groups to examine ataxin-3 function discovered a putative interaction with the human homologs of Rad23, involved in nucleotide excision repair of ultraviolet (UV)-damaged DNA [2]. The interaction of ataxin-3 and FOXO4 highlighted a possible connection to the oxidative stress response. While Gao et al. claim that oxidative stress is not the driving force behind an increase in DNA damage in SCA3, future work should address the specific link between polyQ-ataxin-3 and the oxidative stress response. Furthermore, one crucial piece of missing information is whether the deubiquitination activity of ataxin-3 somehow modulates PKNP function. Despite these remaining questions, these two studies provide an exciting link between polyQ-mediated cerebellar degeneration and neuronal DNA damage. It has long been recognized that mutations in other DNA repair proteins, such as aprataxin and TDP1, result in ataxia and selective cerebellar neurodegeneration [14–16], highlighting the importance of DNA damage repair in the cerebellum. A link between ataxin-3 and PNKP thus reinforces the importance of DNA repair in maintaining healthy cerebellar neurons and suggests targets for therapy development for SCA3 cerebellar degeneration, with possible application to other cerebellar degenerative diseases.
